# The diagnosis and treatment for primary cardiac angiosarcoma with N-ras gene mutation and MSI-L: A case report and review of the literature

**DOI:** 10.1097/MD.0000000000036682

**Published:** 2023-12-22

**Authors:** Jiachun Sun, Tingting Wei, Bo Sun, Jingxiang Su, Hongyan Liu, Dengkui Wang, Xinyang Li

**Affiliations:** a Henan Key Laboratory of Cancer Epigenetics, Cancer Institute, The First Affiliated Hospital, College of Clinical Medicine, Medical College of Henan University of Science and Technology, Luoyang, China; b School of Basic Medical Sciences, Henan University of Science and Technology, Luoyang, China.

**Keywords:** angiosarcomas, immunotherapy, prognosis

## Abstract

**Rationale:**

Primary cardiac angiosarcomas (PCA) is a rare malignancy with a poor prognosis. Currently, there is no standard treatment protocol for the PCA. We report a case of PCA in a 51-year-old woman.

**Patient concerns:**

A 51-year-old woman initially presented with unexplained palpitations and chest tightness accompanied by nausea and vomiting, which worsened after activity and improved after rest. After symptomatic treatment, the symptoms improved, and the above symptoms recurred 8 months later.

**Diagnoses:**

Positron emission tomography-computed tomography revealed multiple lung nodules of varying sizes, some of which exhibited increased glucose metabolism. Furthermore, a soft tissue mass protruding into the pericardial cavity and involving the adjacent right atrium was observed in the right pericardium. The mass exhibited increased glucose metabolism, suggestive of a pericardial tumor with multiple lung metastases. Finally, histopathologic diagnosis of metastatic angiosarcoma was done by computed tomography-guided percutaneous lung and mediastinal biopsy.

**Interventions:**

The patient was treated with palliative chemotherapy for the primary cardiac angiosarcomas and hematogenous lung metastasis. One cycle later, the result of Next-Generation Sequencing showed that the microsatellite instability status was determined to be low-level. Based on this result, tislelizumab was added to the original chemotherapy regimen.

**Outcomes:**

Unfortunately, the patient with PCA passed away after only 2 cycles of chemotherapy, and the cause of death remained unknown.

**Lessons:**

This case report well demonstrates typical imaging findings of a rare cardiac angiosarcomas and emphasizes importance of early investigation for accurate diagnosis and proper management of the cardiac angiosarcomas.

## 1. Introduction

Primary cardiac tumors are rare, accounting for only 0.17% to 0.33% of all cardiac neoplasms. Among cardiac tumors, approximately 20% to 40% are benign, with myxomas and papillary fibroelastomas being the most common.^[1–3]^ The remaining 60% to 80% of tumors are malignant, primarily sarcomas, while primary cardiac angiosarcomas (PCAs) are exceptionally uncommon. At present, the genetic and pathological characteristics of PCAs are not well-defined, and standardized treatment protocols do not exist. Consequently, the clinical outcomes of affected patients tend to be unfavorable, and clinicians have limited experience in terms of managing these tumors.^[4,5]^ In this report, we present a case of a patient with PCA and concurrent lung metastasis who was admitted to our hospital. We also provide a comprehensive review of the pertinent literature to explore the clinical features, imaging findings, differential diagnosis, treatment options and prognosis associated with PCA.

## 2. Case description

The patient was a 51-year-old female who initially presented to our hospital in December 2021 with unexplained palpitations and chest tightness accompanied by nausea and vomiting. The patient’s symptoms worsened with activity and improved with rest. Echocardiography performed at a local hospital revealed significant pericardial effusion and tachycardia, with a heart rate of 117 beats per minute. Chest computed tomography (CT) showed pericardial and bilateral pleural effusion, along with partial collapse of the lower lungs. Subsequent pericardiocentesis and left pleural drainage were performed, which yielded approximately 300 mL of bloody pericardial effusion and 1000 mL of bloody pleural effusion. However, cytological examination and immunohistochemistry of the pericardial and pleural effusions did not reveal the presence of tumor cells. Following the procedures, the patient’s symptoms significantly improved. As such, she declined further investigation and treatment before being discharged.

**Figure 1.**
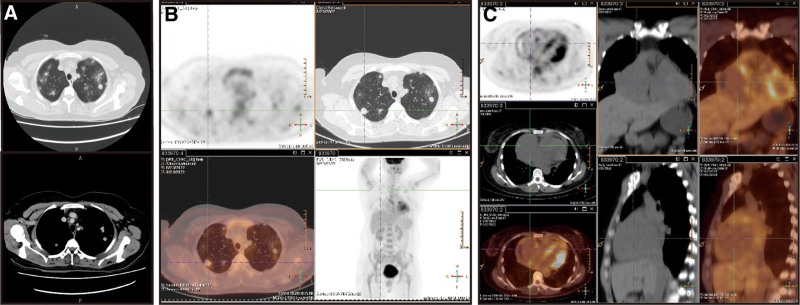
Image examination results. (A) CT showed multiple metastatic nodules in both lungs, with some nodules showing dot halo sign. (B) PET-CT suggested multiple solid nodules of varying sizes in both lungs, with increased glucose metabolism in some lesions, with a maximum SUV value of 3.2. (C) The right pericardium showed a soft tissue mass protruding into the pericardial cavity, with increased glucose metabolism at the margin and a maximum SUV value of about 4.7. CT = computed tomography, PET = positron emission tomography.

**Figure 2.**
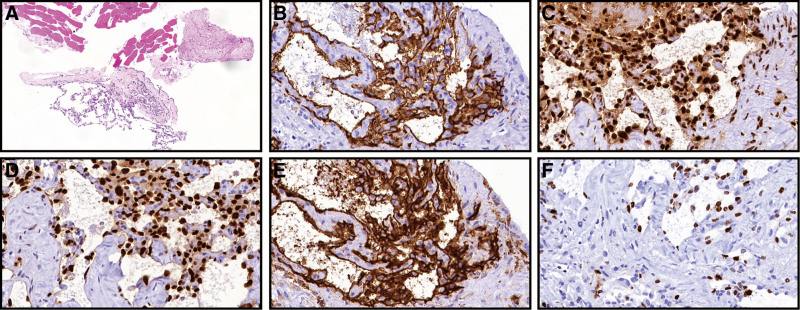
Pathological section of lung metastasis biopsy. (A) The biopsy tissue showed atypical spindle and epithelioid tumor cells, considered for angiosarcoma. (B–F) Immunohistochemistry results, respectively, CD34 (+), FLI-1 (+), ERG (+), CD31 (+), and Ki-67 (60%+).

**Figure 3.**
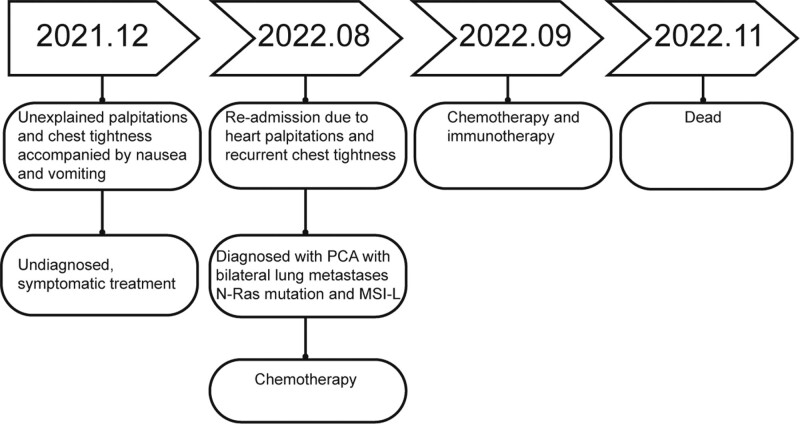
Course and treatment of PCA patient. PCA = primary cardiac angiosarcomas.

In August 2022, the patient was readmitted to our hospital due to recurrence of palpitations and chest tightness. On physical examination, the patient’s heart rate was 119 beats per minute, with sinus rhythm and no audible murmurs. Decreased breath sounds were detected in the right upper lung lobe, while no apparent abnormalities were observed in the jugular veins. No other abnormal findings were observed. Chest CT showed the following: (1) multiple bilateral lung nodules, suggesting metastases with infiltration around some lesions, partial consolidation in the right middle lobe and left upper lobe and enlarged mediastinal lymph nodes; (2) pericardial effusion; (3) a hypodense lesion in segment 3 of the liver (Fig. 1A). Positron emission tomography (PET)-CT revealed multiple lung nodules of varying sizes, some of which exhibited increased glucose metabolism (Fig. 1B). Furthermore, a soft tissue mass protruding into the pericardial cavity and involving the adjacent right atrium was observed in the right pericardium. The mass exhibited increased glucose metabolism, with a maximum standardized uptake value of approximately 4.7, suggestive of a pericardial tumor with multiple lung metastases (Fig. 1C). To obtain a definitive diagnosis, CT-guided percutaneous lung and mediastinal biopsy was performed. The subsequent pathological report indicated angiosarcoma, with the tumor cells exhibiting hobnail morphological features, atypia, and a high proliferation index. The immunohistochemistry results showed the following features (Fig. 2): cytokeratin (CK) (−), epithelial membrane antigen (−), vimentin (+), CD34 (+), CD31 (+), CD68 (−), CK7 (−), thyroid transcription factor-1 (−), P40 (−), S100 (−), D2-40 (−) and Ki-67 (approximately 60%+). Additionally, comprehensive targeted gene testing of the solid tumor revealed the following mutations: *NRAS* Exon 3 c.181c>A p.Q61K with a mutation abundance of 10.89%; *PMS2* Exon 4 c.311T>A p.F104s with a mutation abundance of 56.95%; and *ATM* Exon 17 c.2633 C>T p.T8781 with a mutation abundance of 47.92%. The microsatellite instability status was determined to be low-level (MSI-L). The patient was diagnosed with PCA with bilateral lung metastases. In September 2022, the patient underwent chemotherapy and immunotherapy, specifically paclitaxel (albumin-bound) plus tislelizumab. The administered doses were as follows: paclitaxel (albumin-bound), 400 mg via intravenous infusion; tislelizumab, 200 mg via intravenous infusion. The patient underwent 2 cycles of chemotherapy combined with immunotherapy, but she did not return for the follow-up examinations. In November 2022, during a follow-up with the patient’s family, it was revealed that the patient had passed away, and the cause of death remained unknown. As a result, it was impossible to evaluate the patient’s response to the treatment, which is the defective aspect of this case. The course and treatment flow chart of PCA patients (Fig. 3).

## 3. Discussion

Angiosarcomas typically manifest in various locations, such as the skin, breast, liver, spleen and deep soft tissues, and they account for approximately 1% to 2% of all sarcomas. Notably, nearly half of angiosarcomas occur in the head and neck.^[6,7]^ However, PCA is a rare malignant tumor that specifically originates from the heart. The literature review indicates that only sporadic case reports and retrospective analyses from single centers have been published on this topic. Furthermore, these reports seldom delve into the treatment options and prognosis of cardiac angiosarcoma. The etiology and pathogenesis of PCA remain unclear. Most cases originate from the right atrium, and occurrences in the right ventricle, pericardium or epicardium are rare. Tumors can infiltrate adjacent cardiac structures, including the cardiac walls, pericardium, atrioventricular valves and superior and inferior vena cava, which is often accompanied by the presence of serous effusion (such as pericardial and pleural effusion), as well as clinical manifestations of cardiac tamponade. PCA metastasis is common, with the lungs being the most frequently affected site. Metastases to the lung often present as multiple nodular metastases. Other sites of metastasis include the liver, adrenal glands, spleen and bones, while metastases to the brain are exceptionally rare.^[2,3,8]^

Regarding the patient’s initial presentation, she initially sought care at the neurology department of an external hospital due to dizziness, along with accompanying nausea and vomiting. Brain magnetic resonance imaging (MRI) revealed no abnormal density within the brain parenchyma. Concurrently, cardiac color Doppler echocardiography demonstrated a substantial amount of pericardial effusion and tachycardia. Chest CT further revealed pericardial and bilateral pleural effusion, accompanied by atelectasis in the lower lung lobes. Consequently, the patient underwent pericardial drainage and thoracic tube insertion for drainage. The fluid obtained from both the pericardial and pleural drains was bloody. However, cytological examination of the pericardial and pleural fluid did not reveal the presence of tumor cells. Additional imaging with PET-CT was recommended to the patient to rule out the presence of a tumor, but the patient and her family refused to pursue further investigation. Subsequently, the patient’s symptoms improved, leading to her discharge from the hospital. It is important to note that the patient did not seek any further treatment during this period. However, after about 3 months, the patient was readmitted to our hospital due to symptom recurrence, including palpitations and chest tightness. Subsequent chest CT and whole-body PET-CT revealed the presence of a pericardial tumor, along with multiple lung metastases. To address the symptoms, the patient underwent repeated pericardial and pleural drainage, and cytological examination of the fluid obtained from both sites once again did not reveal the presence of tumor cells. To establish a definitive diagnosis, a CT-guided percutaneous lung and mediastinal biopsy was performed. The results of the postoperative pathological examination confirmed the presence of a malignant tumor originating from the blood vessels, which was consistent with the diagnosis of PCA with lung metastasis. Due to the rarity of this disease, the lack of established treatment protocols and the presence of hemoptysis, targeted therapies commonly used for PCA, such as anlotinib^[9–11]^ or pazopanib,^[12]^ were not administered to the patient. Instead, based on the patient’s symptoms and a review of relevant literature, we initiated treatment with paclitaxel (with albumin) monotherapy. Additionally, next-generation sequencing (NGS) was performed on the biopsy specimen, which did not reveal any significant mutations, but it did indicate MSI-L status. Unfortunately, there were no targeted drugs available for the identified mutations. In an effort to enhance the treatment efficacy, the patient was treated with a combination of paclitaxel and a PD-1 monoclonal antibody, tislelizumab, during the second chemotherapy cycle. However, it is important to note that the patient did not seek timely medical attention during the later stages of the disease. During follow-up, it was discovered that the patient had passed away approximately 1 month after her last visit. The overall survival period from the time of diagnosis to death was approximately 3 months.

PCA, which is characterized by atypical early symptoms, presents a diagnostic challenge. Imaging examinations play a crucial role in the initial assessment of this condition. Techniques such as transesophageal and thoracic echocardiography, cardiac CT and MRI have been extensively used in clinical practice. The increasing popularity of PET-CT in large hospitals has further enhanced its value in early lesion detection, both in terms of primary and metastatic lesions, as well as in monitoring the effectiveness of tumor resection, postoperative radiotherapy and chemotherapy.^[13–15]^ Histopathological diagnosis, however, remains the gold standard for confirming the presence of a tumor. In addition to examining the typical microscopic features, immunohistochemistry markers, such as CD31 (+), CD34 (+), ERG (+) and FVIII (+), are relatively characteristic of PCA.^[16]^ The challenge in pathological diagnosis lies in obtaining standardized tissue samples during the early stages of the lesion.

Currently, there is no established standard treatment regimen for PCA. However, surgical resection combined with radiotherapy and chemotherapy is commonly recommended by experts.^[17]^ Notably, radiotherapy and chemotherapy are limited by the sensitivity of the heart and the risk of radiation-induced lung damage. Therefore, for patients without metastasis, early surgical resection is often recommended as the primary treatment option. However, in many cases, distant metastases have already occurred at the time of diagnosis, resulting in the loss of surgical opportunities in the majority of patients. Furthermore, PCA tends to grow rapidly and infiltrate locally, making the goal of surgery primarily focused on alleviating clinical symptoms. For patients who are not suitable candidates for surgical resection and who do not have metastasis, heart transplantation may be considered as a treatment option. However, it should be noted that the use of immunosuppressants following heart transplantation can increase the risk of tumor recurrence and metastasis. The study conducted by Look Hong^[7]^ demonstrated that regardless of the chosen treatment method, the overall average survival time for PCA ranges from 5 to 17 months. Therefore, the key to improving the prognosis of PCA lies in early diagnosis and effective drug development. Exploring tumor markers that facilitate early diagnosis and identifying new targets for drug treatment are crucial in this regard. Particularly, the emergence of immune checkpoint inhibitors holds promise for the treatment of advanced PCA.

Upon reviewing the diagnosis and treatment process of this patient, it is evident that the disease progressed rapidly, leading to an extremely poor prognosis. The total duration from the onset of clinical symptoms to the final declaration of clinical death was 11 months, with a particularly short span of 3 months from the diagnosis of PCA with multiple lung metastases to the declaration of clinical death. This highlights the importance of early diagnosis and treatment to achieve better clinical outcomes. This case brings our attention to several important considerations. First, early diagnosis and treatment are crucial for better clinical outcomes. It is challenging to obtain a definitive diagnosis for suspected PCA solely based on clinical characteristics and imaging findings. Therefore, there is an imperative need to establish a safe and efficient biopsy technique specifically for cardiac tumors. Second, the patient experienced recurrent cough and hemoptysis, indicating a bleeding tendency. Although anlotinib has shown efficacy in the treatment of soft tissue sarcomas, including angiosarcomas, its use and potential risks need further discussion and consideration. Third, the patient’s NGS results revealed mutations in the *NRAS, PMS2* and *ATM* genes. Although there are currently no targeted therapies available for these specific mutations, advancements in diagnostic and therapeutic techniques, along with molecular biology technology, hold promise for the development of targeted therapies for primary cardiac sarcomas. Fourthly, the patient showed a low MSI, and although the use of the PD-1 monoclonal antibody did not demonstrate significant efficacy, the potential of immune checkpoint inhibitors in the treatment of primary cardiac sarcomas cannot be disregarded. Finally, although this study objectively, truthfully, and completely records the diagnosis and treatment of a case of primary cardiac angiosarcoma, as a case report, it cannot represent the entirety of patients with this condition. Therefore, future work should involve comprehensive literature reviews to summarize published cases and related studies, identifying clinical characteristics of the disease. Additionally, collaboration with other institutions to conduct randomized, controlled studies is essential for accumulating research data on this condition.

## 4. Conclusion

In summary, PCA is a rare disease with a low incidence rate and nonspecific early symptoms. In the majority of cases, diagnosis often occurs when metastases are already present. Standard treatment regimens are lacking, and the prognosis is generally poor. Therefore, further research is needed in the areas of diagnosis, differential diagnosis, molecular targeted therapy, immune checkpoint inhibitors and prognostic assessment to improve disease management.

## Author contributions

**Conceptualization:** Jiachun Sun, Dengkui Wang, Xinyang Li.

**Data curation:** Tingting Wei, Bo Sun.

**Investigation:** Jiachun Sun, Jingxiang Su, Hongyan Liu.

**Writing – original draft:** Jiachun Sun, Xinyang Li.

**Writing – review & editing:** Jiachun Sun, Xinyang Li.
